# Modeling the Population Health Impact of Introducing a Modified Risk Tobacco Product into the U.S. Market

**DOI:** 10.3390/healthcare6020047

**Published:** 2018-05-16

**Authors:** Smilja Djurdjevic, Peter N. Lee, Rolf Weitkunat, Zheng Sponsiello-Wang, Frank Lüdicke, Gizelle Baker

**Affiliations:** 1 Philip Morris International R&D, Philip Morris Products S.A., Quai Jeanrenaud 5, Neuchâtel 2000, Switzerland; Smilja.Djurdjevic@pmi.com (S.D.); Rolf.Weitkunat@pmi.com (R.W.); Zheng.Wang@pmi.com (Z.S.-W.); Frank.Luedicke@pmi.com (F.L.); 2 P. N. Lee Statistics and Computing Ltd., 17 Cedar Road, Sutton SM2 5DA, UK; PeterLee@pnlee.co.uk

**Keywords:** public, tobacco, risk, modified, reduced, nicotine, non-combustible, health, smoking, harm

## Abstract

Philip Morris International (PMI) has developed the Population Health Impact Model (PHIM) to quantify, in the absence of epidemiological data, the effects of marketing a candidate modified risk tobacco product (cMRTP) on the public health of a whole population. Various simulations were performed to understand the harm reduction impact on the U.S. population over a 20-year period under various scenarios. The overall reduction in smoking attributable deaths (SAD) over the 20-year period was estimated as 934,947 if smoking completely went away and between 516,944 and 780,433 if cMRTP use completely replaces smoking. The reduction in SADs was estimated as 172,458 for the World Health Organization (WHO) 2025 Target and between 70,274 and 90,155 for the gradual cMRTP uptake. Combining the scenarios (WHO 2025 Target and cMRTP uptake), the reductions were between 256,453 and 268,796, depending on the cMRTP relative exposure. These results show how a cMRTP can reduce overall population harm additionally to existing tobacco control efforts.

## 1. Introduction

Worldwide, there are approximately 1.1 billion smokers, and nearly 6 million deaths are attributed to smoking annually [1]. A number of serious diseases are associated with tobacco smoking that result in smoking-related mortality, including cardiovascular diseases, lung cancer, and chronic obstructive pulmonary disease (COPD). For many years, preventing smoking initiation and promoting smoking cessation were the primary strategies for reducing the harm associated with cigarette smoking. Although smoking prevalence has declined over the last 40 years, those declines have flattened in many countries in the last 10 years [1].

Smoking cessation is clearly the most effective strategy for smokers to reduce their risk of harm and disease. However, the number of former smokers who relapse is high. Approximately 80% of smokers who attempt to quit smoking return to smoking within one month, and annually, approximately 5% of smokers quit successfully [2]. Philip Morris Internationsl (PMI) is developing non-combustible tobacco and nicotine containing products that have the potential to present less risk of harm to smokers who switch to these products versus continued smoking. We refer to these products as candidate modified risk tobacco products (cMRTPs). Harm reduction is, by definition, a strategy used in medicine and social policy to minimize harm to individuals and/or wider society from hazardous behaviors or practices that cannot be completely avoided or prevented [3].

In this context, a complementary approach to existing strategies to reduce smoking prevalence is starting to gain support from a range of stakeholders, including public health organizations, healthcare professionals, and regulators. According to the Royal College of Physicians, embracing such an approach could offer a means to prevent millions of deaths [4] and to hasten our progress to a tobacco-free society [3].

In 2012, the U.S. Food and Drug Administration (FDA) released a draft guidance for MRTP applications that described the evidentiary requirements for claims of reduced exposure and reduced risk [5]. An cMRTP is defined by the U.S. Family Smoking Prevention and Tobacco Control Act as ”any tobacco product that is sold or distributed for use to reduce harm or the risk of tobacco related disease associated with commercially marketed tobacco products.” At PMI, cMRTP is the term used to refer to products that present, are likely to present, or have the potential to present less risk of harm to smokers who switch to these products versus continued smoking. PMI has a range of cMRTPs in various stages of development, scientific assessment, and commercialization. Because the cMRTPs do not burn tobacco, they produce far lower quantities of harmful and potentially harmful compounds than found in cigarette smoke.

In 2016, PMI filed an MRTP application for its Tobacco Heating System (THS) [6], with aerosol containing lower levels of harmful and potentially harmful toxicants than found in cigarette smoke, thus offering reduced exposure to these toxicants, reduced toxicity, and reduced health risks to consumers compared with continued cigarette smoking. In 2017, the Committee on Toxicology (COT) released a statement that THS investigations show a decrease in the harmful and potentially harmful constituents (HPHC), of approximately 50% for some HPHCs and more than 90% for others, with many constituents below the limits of detection in the aerosol generated by the device compared to smoke from a conventional cigarette [7].

Population harm reduction depends both on the availability of lower-risk products and on a significant number of adult smokers being willing to accept and switch to these products. Conversely, low product acceptance could theoretically offset even substantial, product-specific risk reduction. Concerns have been raised that the benefits of alternative products could be offset by an increase in unintended consequences, such as smoking initiation, interference with quitting among smokers, or encouraged re-initiation in former smokers.

One difficulty is that population-level data on consumer product use and behaviors cannot be collected prior to marketing a cMRTP, so the impact of marketing the product on the health of the population as a whole cannot be assessed fully. Therefore, PMI has implemented a comprehensive pre-market product assessment program [8] based on FDA’s 2012 draft Guidance [5] as well as an applicable post-market research program to assess the impact of marketing products on the population as a whole.

Initially, this model will use assumptions to predict what may happen following the launch of the product. However, data from the post-market assessment program can serve as input into the model (replacing the initial assumptions with actual market-derived data) and can also be used to refine the assumptions moving forward.

PMI has developed the Population Health Impact Model (PHIM) to quantify, in the absence of epidemiological data, the effect that marketing a cMRTP may have on the health of the population as a whole [9,10]. This activity is accompanied by an increasing number of publications on alternative modeling approaches for estimating the population health impact following changes in the nicotine and tobacco product landscape [11,12,13,14]. The FDA acknowledges the inherent difficulties of such models, as they require assumptions about how today’s consumers, both users and non-users of tobacco products, will modify their future behavior in response to the market entry of an cMRTP. Therefore, it is of key importance to describe these assumptions, and how variation in them affects the estimated population health impact, clearly.

## 2. Materials and Methods

The methodology of the PHIM has been described in detail [9] and was designed to assess the population-level health impact of marketing a cMRTP as a function of the risk or toxicity of the product to the individual user and the product use prevalence distribution in the population. It involves comparison of mortality from the four main smoking-related diseases (lung cancer, COPD, ischemic heart disease and stroke) in two groups of individuals who, at the start of the defined follow-up period, each have a distribution of smoking habits that is identical, and representative of the country and sex considered at baseline. The populations are then followed up under two scenarios, a “null scenario” in which the cMRTP is not introduced and an “alternative scenario” in which it may be introduced, with “transition probabilities” determining the rate at which individuals change smoking groups. Based on the tobacco use histories built up, the relative risks of each disease are then estimated for each individual, and used to determine the number of deaths attributable to tobacco. The difference between the estimated numbers of deaths for the two scenarios then gives the difference in mortality associated with the introduction of the cMRTP.

More details of the method are given below. The code will in due course be made publically available on the internet.

### 2.1. Estimating Tobacco Use Histories

In the application of the PHIM described here, simulated samples of 10,000 males and 10,000 females start aged 10–79 in 1990 with a distribution of smoking habits (never, current or former smokers) and time of quit among never smokers that is representative of the U.S. at that time. It is assumed that cMRTP had not yet been introduced by 1990. For both the null and the alternative scenarios individual tobacco histories are then updated each year until 2010 based on defined transition probabilities. In the null scenario, the tobacco groups remain as never, current, and former smokers, and the transition probabilities are set so as to produce smoking prevalence distributions comparable to those observed in the U.S. In each alternative cMRTP scenario, described below, differing sets of probabilities are used. Where an alternative scenario involves the introduction of a cMRTP, there are five tobacco groups: never tobacco users, current cigarette smokers, current cMRTP users, current dual users, and former tobacco users. Note that any individual reaching age 80 drops out of the population, so by 2010, smoking prevalence refers to those aged 30–79.

### 2.2. Estimating Relative Risks

Separately for the null and alternative scenarios, the PHIM then derives estimates of the relative risk (compared to never tobacco users) for lung cancer, COPD, ischemic heart disease, and stroke for every individual of each sex at each year of follow up. The estimation uses a modification of the negative exponential model which allows for multiple changes in smoking habits. The model requires knowledge of each individual’s tobacco use at each year. It also requires disease and age-specific estimates of the relative risk associated with continued smoking and of the quitting half-life, the half-life being the time after quitting when the increase in relative risk associated with smoking has halved. These estimates, derived from published meta-analyses, are provided in of our earlier paper [10] which clarifies the sources used. The model also requires estimates of the “relative exposure” (RE) corresponding to the current tobacco use pattern. This takes the value 0 for an individual not using tobacco (a never or former user), 1 for a current cigarette smoker, *f* for a current cMRTP user (the *f*-factor), and (1 + *f*)/2 for a dual user, a dual user being an individual whose tobacco use pattern consists of a substantial use of both products, cigarettes and cMRTPs. At the population level, for a given scenario, sex, disease, and follow-up year, the PHIM then estimates the mean relative risk (¯RR) for individuals in each five-year age group from 30–34 to 75–79.

As described elsewhere [10], the method of estimating the relative risk for an individual by age for each disease depends on first calculating what is termed an equivalent dose (ED) at each age, and then multiplying this by the excess relative risk for a continuing cigarette smoker of that age. ED starts at 0, as no individual uses tobacco at birth. As a switch occurs, ED gradually increases towards the RE value for the product switched to. Subsequently, as RE increases (or decreases) at a further switch, ED gradually increases (or decreases) towards the new RE value. More formally, if, at age *a*, RE(*a*) is the relative exposure and H(*a)* is the half-life, one first calculates the negative exponential factor for a single year as

*N*(*a*) = exp (−log_e_(2)/*H*(*a*)), (1)

and then, with ED(1) taken as 0, one calculates subsequent values of ED by the formula

*ED*(*a*) = *N*(*a*) *ED*(*a* − 1) + (1 − *N*(*a*))*RE*(*a*). (2)

### 2.3. Estimating Deaths Attributable to Tobacco Use

To estimate the absolute numbers of deaths attributable to tobacco product use for each scenario in those aged 30–79 years, published U.S. sex- and age-specific numbers of deaths for each of the four diseases are multiplied by (¯RR–1)/¯RR, and the total number of smoking attributable deaths (SADs) from the four diseases combined are obtained by simple addition. The population health impact is then derived by subtracting the numbers under the alternative scenario from those under the null scenario. As equal population size is assumed under both scenarios, an adjustment can be made for the decrease in death rates in the alternative scenario [9,10]. However, the simulations presented in this paper do not use this adjustment, as it had a small effect and did not affect the overall picture.

### 2.4. Null Scenario

The derivation of the smoking histories under the null validation scenario uses the set of transition probabilities described in our earlier publication [10]. That publication demonstrates that there was a reasonably good fit between the smoking distributions generated by PHIM across all these ages in 1990 and 1995, though the estimates for the older age groups do not correspond as strongly with the International Smoking Statistics (ISS) data. Exact correspondence between these two estimates was not expected for various reasons, such as variation due to the simulation process, inaccuracies in the ISS estimates, and ISS estimates for the U.S. population not being available past 2005 (so that 2005 estimates were used for later years).

Estimates of SADs derived from the null scenario have been compared previously with estimates published by the U.S. Surgeon General [15] and in the Morbidity and Mortality Weekly Report [16]. The results for most diseases were similar when compared with the Surgeon General estimates [10]. However, there is a notable difference for COPD, with the null scenario estimates much lower in both sexes. This difference mainly arises due to the much lower current smoking ¯RR estimate of 4.56 for both sexes incorporated in the PHIM. The other estimates were higher, particularly for 2005–2009 [15], assumed as 29.69 at age 65–74 and 23.01 at age 75+ for males and 38.89 at age 65–74 and 20.96 at age 75+ for females. Given that our estimate was based on a published meta-analysis [10] involving 39 North American studies, with the 95% confidence interval for our estimate as narrow as 3.69 to 5.62, it seems likely that the Surgeon General's ¯RR estimate is far too high.

### 2.5. Alternative Scenarios

Eight different alternative scenarios have been used. Some estimate the health impact of introducing a cMRTP onto the market, while others illustrate the effect of alternative methods of risk reduction for comparative purposes.

#### 2.5.1. No Further Smoking

As smoking cessation is the “gold standard” for the maximum risk reduction that can be achieved, this scenario is one in which all current smokers immediately stop smoking, with no further initiation or re-initiation of tobacco use.

#### 2.5.2. Smoking Totally Replaced by cMRTP Use

This scenario illustrates the maximal impact of introducing a cMRTP into the U.S. market. Here, all current smokers in 1990 immediately switch to the cMRTP. Unlike the first alternative scenario, in which initiation and re-initiation are eliminated, it is assumed here that initiation, re-initiation, and quitting rates are the same as in the null scenario but only involve switches to or from the cMRTP. To understand the potential impact of the cMRTP a range of *f*-factors between *f* = 0.1 and *f* = 0.3 is generally considered. In order to derive estimate the *f*-factor, the non-clinical and clinical data collected during the product assessment programs were examined. PMI developed a multivariate*f*-factor distribution based on objective Bayesian statistics using the set of biomarkers of exposure and clinical risk endpoints collected in the studies [17]. In the absence of data to inform the model, we assumed that the *f*-factor is distributed uniformly between 0 (smoking cessation) and 1 (continued smoking) and normality of the conditional distribution of each biomarker given the product use. The relative changes in the parameters provide information on the *f*-factor using a link function of an unknown, multi-endpoint biomarker that ensures the sufficiency of the selected set of biomarkers. The *f*-factors, which are derived from aerosol chemistry and biomarker data, are intended to indicate the highest (90%) and medium (70%) plausible reductions in relative exposure for the cMRTP.

#### 2.5.3. WHO 2025 Target

The World Health Organization’s (WHO) 2015 “Global Report on Trends in Tobacco Smoking 2000–2025” [18] states,

“If the 194 WHO Member States collectively achieved a 30% reduction from the 2010 level of 22.1%, they would be expected to reach a prevalence level of 15.4% in 2025”

(i.e., they target a 30% reduction in the prevalence of tobacco smoking between 2010 and 2025). To examine the impact that such a reduction in smoking prevalence might have on the health of the U.S. population, the third scenario used a set of transition probabilities aimed at reducing smoking prevalence by 30% between 1990 and 2005, with the smoking prevalence being allowed to continue to decline over the last five years of the follow up.

#### 2.5.4. WHO 2025 Projection

The WHO report referenced above goes on to state, “At this stage, it is projected that the prevalence level in 2025 will be 18.9%, or 3.5 percentage points above the target. This would represent a 14% relative reduction overall.” The fourth scenario uses transition probabilities aimed at reducing smoking prevalence by 14% between 1990 and 2005.

#### 2.5.5. cMRTP Uptake Case

For this scenario, the transition probabilities were designed so that within 10 years of marketing the product, 17% of the smoking population uses the cMRTP (15% cMRTP users and 2% dual users), as if the cMRTP was launched in 1990. We aimed for the target but continued to simulate over the next 10 years of follow up.

#### 2.5.6. cMRTP Uptake Case in Addition to the WHO 2025 Target

This scenario examines the effects of combining a 30% reduction in smoking prevalence in 15 years with the addition of 17% of the remaining adult smokers using an cMRTP within 10 years, with both continuing to decline at the same rates for the remainder of the 20-year period.

#### 2.5.7. cMRTP Uptake Case in Addition to the WHO 2025 Projection

Similarly, this scenario examines the effects of combining a 14% reduction in smoking prevalence in 15 years with the addition of 17% of the remaining adult smokers using a cMRTP within 10 years.

#### 2.5.8. Extreme Increase in Dual Use

In this scenario, majority of cMRTP use occurs in a dual use pattern (12.5% of dual users and 5% of cMRTP users) within 10 years of marketing the cMRTP.

The sets of transition probabilities used in the different scenarios can be found in Supplemental file.

## 3. Results

In the application of the PHIM described here, simulated samples of 10,000 males and 10,000 females start aged 10–79 in 1990 with a US-representative distribution of smoking prevalence, and no use of a cMRTP. Individual tobacco histories are then updated each year until 2010 based on two alternative sets of estimated “transition probabilities” of switching between tobacco groups, referred to as “scenarios”. The null scenario and various alternative scenarios are described fully in Section 4. The results for each scenario are described below.

### 3.1. No Further Smoking

**Figure 1:**
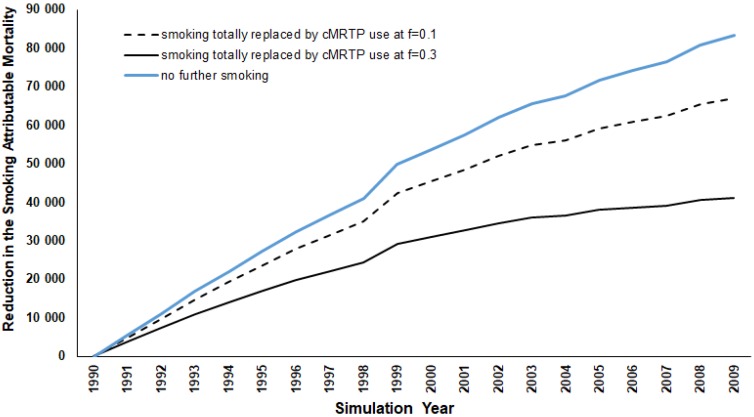
Annual reduction in SADs from 1990 to 2009 for alternative for alternative scenario 1, “No further smoking,” and scenario 2 “Smoking totally replaced by cMRTP use at *f* = 0.1 and *f* = 0.3.” For all years combined, the reductions are 516,944 and 780,433 deaths. The line for the “no further smoking “scenario is superimposed.

While the smoking prevalence remains at zero throughout the 20-year follow-up period, the risks of the four smoking-related diseases following smoking cessation do not diminish instantaneously; instead, the decline is gradual over time, at a rate that is dependent on the disease-specific half-life of excess risk. As shown in Figure 1, the initial annual reduction in SADs is relatively small (approximately 5500 for both sexes and all ages combined in 1991), but this increases annually as the excess risk declines. By the end of the period, in 2009, the annual reduction is over 83,000 per year. Over the 20-year period combined, the total elimination of smoking is estimated to result in 934,947 fewer SADs. Clearly, this number would have continued to increase sharply had follow up been continued for more than 20 years. In the sections that follow, we compare this estimate of the maximal effect achievable in 20 years (934,947 total SADs) with those from other alternative scenarios.

### 3.2. Smoking Totally Replaced by cMRTP Use

**Figure 2:**
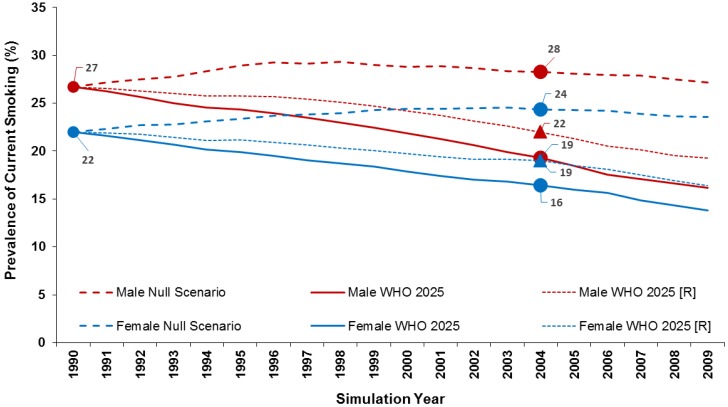
Reduction in smoking prevalence between 1990 and 2005 under the null scenario and the WHO 2025 projections (WHO 2025 reflects the initial estimates of a 30% reduction, WHO 2025 (R) reflects the revised projections).

As in the first scenario, the initial years show a marginal reduction in SADs, between 3726 (*f*-value = 0.30) and 4907 (*f*-value = 0.10) in 1991 (see Figure 2). However, by the end of the period, the introduction of the cMRTP resulted in a cumulative reduction in SADs between 516,944 and 780,433 (55% to 83% of the results seen for no further smoking).

### 3.3. WHO 2025 Target

At baseline in 1990, smoking prevalence was 26.7% in males and 22.0% in females. In the null scenario, smoking prevalence (at ages up to 79 years) remained relatively constant over the 20-year period—26.8% in males and 23.5% in females in 2009. In the WHO 2025 Target scenario (30% reduction in 15 years), the transition probabilities produced smoking prevalence in 2005 of 18.5% in males and 16% in females. These were equivalent to 30.7% and 27.3% reductions, respectively, quite close to the 30% reductions the WHO sought to achieve. This scenario resulted in a cumulative total of 172,458 fewer SADs over the 20-year period (108,637 in males and 63,820 in females).

### 3.4. WHO 2025 Projection

**Figure 3:**
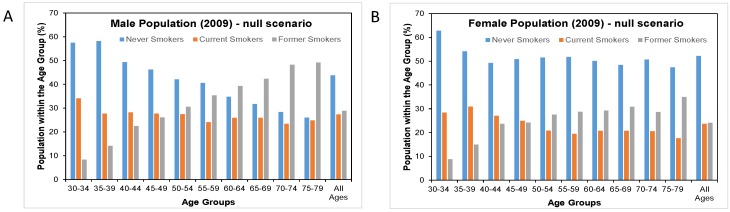

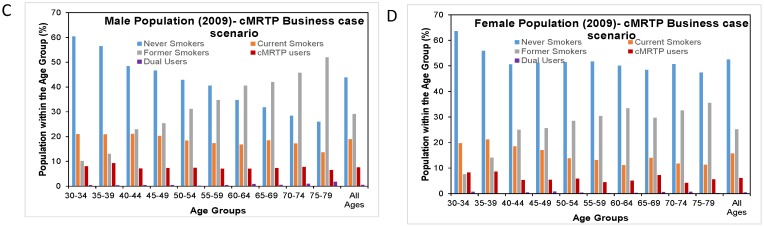
Distribution of smoking patterns by age and sex in 2009 in the null scenario and in the cMRTP uptake case scenario. (**A**) is the distribution of smoking patterns by age in the male population under the null scenario while (**C**) is the same male population under the Business case scenario. (**B**) is the distribution of smoking patterns by age in the female population under the null scenario while (**D**) is the same female population under the Business case scenario.

In the WHO 2025 Projection scenario (14% reduction in 15 years), the transition probabilities used produced smoking prevalence of 21.3% in males and 18.5% in females in 2005. These were equivalent to 20.2% and 15.7% reductions, respectively, somewhat greater than the 14% reduction they were designed to produce. Here, there was a cumulative total reduction of 111,102 fewer SADs (69,042 in males and 42,060 in females). Figure 3 illustrates the trends in smoking prevalence in the two WHO scenarios as compared to the null scenario.

### 3.5. cMRTP Uptake Case

**Figure 4:**
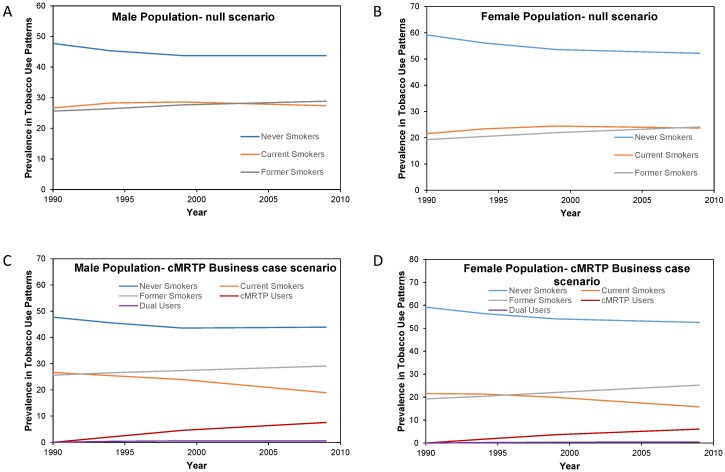
Change in smoking prevalence patterns by sex over the period 1990 to 2009 in the null scenario and in the cMRTP uptake case scenario by sex. (**A**) is the change in smoking prevalence over time in the male population under the null scenario while (**C**) is the change in smoking prevalence for the same male population under the Business case scenario. (**B**) is the change in smoking prevalence over time in the female population under the null scenario while (**D**) is the change in smoking prevalence for the same female population under the Business case scenario.

Here, there was very little change in the prevalence of never smokers and former smokers between the null scenario and the cMRTP scenario, as the majority of cMRTP users and dual users transitioned to this state from the cigarette smoking state. By the end of follow up, the initial smoking prevalence of 26.6% in males had become 18.3% current cigarette smokers, 7.9% cMRTP users, and 0.5% dual users. In females, 21.6% of initial smokers had become 15.3% current cigarette smokers, 6.2% cMRTP users, and 0.5% dual users (see Figure 4).

In this scenario, there were 90,156 fewer cumulative SADs up to 2009 for an *f*-value of 0.1 and 70,275 fewer for an *f*-value of 0.3. In an additional alternative in which the rates of switching were the same but the cMRTP was assumed to have an *f*-value of 0, there were 100,235 fewer cumulative SADs (67,151 in the male population and 33,083 in the female population). This is the case in which the same consumers were switched to smoking cessation.

### 3.6. cMRTP Uptake Uase in Addition to the WHO 2025 Target

In this case, the modeling simulation resulted in between 256,453 and 268,796 fewer SADs over the 20-year period for a cMRTP with an *f*-factor between 0.1 and 0.3.

### 3.7. cMRTP Uptake Case in Addition to the WHO 2025 Projection

Here, the combination of tobacco prevention and cMRTPs resulted in estimated reductions in cumulative total SADs of 186,876 (*f* = 0.1) and 170,026 (*f* = 0.3). This scenario and the previous one demonstrate how cMRTPs such as the THS can complement existing efforts to reduce SADs.

### 3.8. Extreme Increase in Dual Use

In this scenario, after 10 years, there is still 17.5% of cMRTP use in the smoking population. However, 5% are mainly using cMRTPs, while 12.5% are dual users. Despite this increase in the prevalence of dual use, simulation results show reduction in cumulative total SADs between 45,802 and 59,840 over the 20-year period for an *f*-value between 0.1 and 0.3.

To summarize the above, Table 1 summarizes smoking prevalence at the end of the 20-year period for the various alternative scenarios, while Table 2 compares the reductions in cumulative SADs. Fuller details of the trends in smoking prevalence can be found in Supplemental file. 

## 4. Discussion

Clearly, cessation brings the greatest benefits to the health of a population as a whole and can result in a significant number of lives saved, with 934,947 fewer SADs estimated to occur in the U.S. upon total elimination of smoking after 20 years. This number will increase further in subsequent years. Although this is an extreme scenario and very unlikely to become reality, it has been designed and tested to demonstrate the maximum potential risk reduction for the U.S. population, and it can be considered a point of reference for every other scenario investigated.

In scenarios where a cMRTP is introduced to the U.S. population, the extreme case, in which smoking is immediately and totally replaced by cMRTP use, produced reductions in SADs that were 55% to 83% of that for total cessation, depending on the cMRTP relative exposure. In scenarios that are less extreme and could become reality, reductions were less marked but still important and relevant. The WHO 2025 Projection of a 14% reduction in smoking prevalence in 15 years can lead to 3.1% fewer SADs in the U.S. over a 20-year period. If cMRTPs are introduced into the market as defined in the cMRTP uptake case, this percentage will increase 4.7% to 5.2% further depending on the *f*-factor for the cMRTP. Introducing cMRTPs similarly increases the reductions in prevalence in line with the WHO 2025 Target.

Overall, in every scenario considered, whether it involves complete or partial elimination of smoking or a replacement of some or all smokers with cMRTP users, a benefit on population health is shown, as quantified by a reduction in SADs. Even the extreme increase of dual use scenario, which can be considered as the worst scenario given the increased relative exposure for dual use (*f*-value range 0.55 to 0.65) as compared with cMRTP use (*f*-value range 0.1 to 0.3), resulted in a small reduction in SADs, and the higher the prevalence of dual use, the less SADs decline will be observed. Therefore, it is important that consumers of cMRTPs understand that the product is more effective when is not combined with cigarettes.

The negative exponential model we use to estimate relative risks from tobacco histories would benefit from the large study data collecting extensive information on changes in tobacco use over time as a part of ongoing refinement process. 

The model assumes that individuals only smoke cigarettes and/or use cMRTPs and does not account for other forms of tobacco use. Smokeless tobacco and nicotine replacement therapy are believed to have little or no effect on the risk of the diseases studied. Ignoring e-cigarettes may also not be important, if claims [19] that any health effects are less than 5% of those from cigarettes are correct. Ignoring cigar and pipe smoking may seem more relevant. The model effectively assumes that cigar and pipe smoking carry the same risk as cigarette smoking, as the initial smoking status of the populations followed is based on the prevalence of smoking any product rather than only cigarettes. Had we allocated initial smoking status based on estimates of prevalence of cigarette smoking, we would instead have effectively assumed that cigar and pipe smokers have the same risk as never smokers, which would clearly be a less appropriate assumption. The problem, of course, is that extending the tobacco groups to include pipe and cigar smokers (and possibly also cigarette smokers with differing consumption levels) would require estimation of a huge number of transition probabilities, which would be difficult or impossible to do reliably. In the context of the U.S., our treatment of pipe and cigar smoking is probably unimportant, as cigarette smokers form the vast majority of all smokers.

The 30% and 14% reductions referred to by the WHO are presumably related to populations of the same or similar age, and we have applied them in situations where the simulated population is ageing. Given that in the null scenario, smoking prevalence (at ages up to 79 years) remained relatively constant over the 20-year period, the results we have produced in scenarios 3, 4, 6, and 7 should still provide a good illustration of the effects of the different scenarios. The fact that the chosen set of transition probabilities produces reductions in smoking prevalences that did not exactly align with the WHO reductions also seems unimportant for analyses that are intended to provide a broad indication of the relative effects of the alternative scenarios.

We do not account for environmental tobacco smoke (ETS) exposure, where we showed earlier [9] that whether or not the cMRTP reduces the risk from ETS exposure would have little effect on the estimated drop in mortality associated with cMRTP introduction.

In the absence of reliable available estimates on relative risk and half-lives for all of the diseases associated with smoking, we have limited attention to the four major smoking-associated diseases. We estimated earlier [9] that overall estimates of deaths saved due to the introduction of the cMRTP would have to be increased by approximately 50% to give an estimate for all smoking-related diseases combined. Increasing our estimates of deaths by 50% should give a reasonably accurate estimate for all smoking-related diseases unless the average rate of decline in risk following quitting differs markedly between the diseases we have and have not considered.

Our estimates of deaths saved may be in error if those who switch from cigarette smoking to cMRTP use tend to be atypical in some ways (e.g., having a duration of smoking markedly different from those who do not switch) or change their distribution of other risk factors (e.g., their degree of alcohol consumption).

More importantly, our analyses have limited attention to a 20-year follow up, particularly if the introduction of the cMRTP has an effect on the initiation of tobacco use by adolescents and young men and women. While the increase in prevalence would be clear after 20 years, any effect on mortality would be minimal, as the great majority of the additional tobacco users would be less than 50 years old.

Despite these reservations, we believe that the results summarized here provide a good insight into the extent to which introduction of a cMRTP might affect the distribution of tobacco use and the number of deaths associated with tobacco and clarify the assumptions that are most critical to the predictions.

The closeness of the null scenario predictions to actual epidemiological and authoritative statistics from the U.S. population across the 20-year study period provides a solid basis for assessing the potential population benefit of a cMRTP [10]. As shown in that paper, the introduction of a cMRTP would result in fewer SADs in all but the most unlikely situations. The degree to which SADs are reduced is influenced primarily by the prevalence of cMRTP use, maximized in the scenario in which there is complete switching by adult smokers with no influence on non-smokers. In the real world, consumers will need to understand the relative health benefits of switching from cigarettes to cMRTPs. It will take time to determine this precisely, as a meaningful number of smokers will first have to convert to cMRTPs. During this time, it will be important to conduct post-market surveillance and studies that would provide additional insights and encourage switching behavior among smokers.

## 5. Conclusions

Overall, based on the scenario assumptions within the various PHIM simulations, introducing a cMRTP into the U.S. population will lead to a net public health benefit in terms of reduced tobacco-related mortality.

## Figures and Tables

**Table 1:** Summary of smoking prevalence after the 20-year period for the null and alternative scenarios.

Modeling Scenario	Never Smokers (%)	Current Smokers (%)	cMRTP Users (%)	Dual Users (%)	Former Smokers (%)
**Males, all ages**					
Null scenario	44.0	27.6	0.0	0.0	28.3
1. No further smoking	52.3	0.0	0.0	0.0	47.7
2. Smoking replaced by cMRTP	44.1	0.0	27.4	0.0	28.4
3. WHO 2025 Target	45.8	15.7	0.0	0.0	38.5
4. WHO 2025 Projection	45.8	18.8	0.0	0.0	35.4
5. cMRTP uptake case	44.1	18.4	7.9	0.6	29.1
6. cMRTP uptake + WHO Target	43.2	10.3	5.0	0.0	41.5
7. cMRTP uptake + WHO Projection	45.8	13.5	5.4	0.4	35.0
8. Extreme increase in dual use	43.9	21.0	2.3	4.2	28.6
**Females, all ages**					
Null scenario	52.3	23.7	0.0	0.0	24.0
1. No further smoking	60.2	0.0	0.0	0.0	39.8
2. Smoking replaced by cMRTP	52.6	0.0	23.6	0.0	23.8
3. WHO 2025 Target	54.4	13.3	0.0	0.0	32.3
4. WHO 2025 Projection	54.4	16.1	0.0	0.0	29.6
5. cMRTP uptake case	52.6	15.4	6.2	0.5	25.4
6. cMRTP uptake + WHO Target	52.1	8.6	4.5	0.0	34.9
7. cMRTP uptake + WHO Projection	54.4	11.3	4.4	0.3	29.5
8. Extreme increase in dual use	52.5	17.7	1.9	3.4	24.4

The null scenario values are the same or very similar for all eight modeling scenarios.

**Table 2:** Reduction in cumulative SADs for the various alternative scenarios (data for both sexes and the four diseases combined).

Modeling Scenario	Cumulative SADs	*f*-Factor	Reduction in Cumulative SADs	% Drop in SADs
Null scenario	3,581,652	-	-	-
Alternative scenarios	-	-	-	-
1. No further smoking	-	NA	934,947	26.1
2. Smoking replaced by cMRTP	-	*f* = 0.1	780,433	21.8
		*f* = 0.3	516,944	14.4
3. WHO 2025 Target	-	NA	172,458	4.8
4. WHO 2025 Projection	-	NA	111,102	3.1
5. cMRTP uptake case	-	*f* = 0.1	90,155	2.5
		*f* = 0.3	70,274	2.0
6. cMRTP uptake + WHO Target	-	*f* = 0.1	268,796	7.5
		*f* = 0.3	256,453	7.2
7. cMRTP uptake + WHO Projection	-	*f* = 0.1	186,876	5.2
		*f* = 0.3	170,026	4.7
8. Extreme increase in dual use	-	*f* = 0.1	59,840	1.7
		*f* = 0.3	45,802	1.3

## Supplementary Materials

The following are available online at http://www.mdpi.com/2227-9032/6/2/47/s1.
