# Supplementary material for: Modeling the Population Health Impact of Introducing a Modified Risk Tobacco Product into the U.S. Market
Source: Healthcare (Basel). 2018 May 16;6(2):47. doi: 10.3390/healthcare6020047 (PMC6023310; doi:10.3390/healthcare6020047)
Supplement: Supplementary file 1 [file healthcare-06-00047-s001.pdf]

healthcare

Supplemental Materials

Modeling the Population Health Impact of Introducing a Modified Risk Tobacco Product into the U.S. Market

Smilja Djurdjevic, Peter N. Lee, Rolf Weitkunat, Zheng Sponsiello-Wang, Frank Lüdicke and Gizelle Baker

The Sets of Transition Probabilities Used in the Different Scenarios

For the null scenario, the probability of transition between the three states (N = never, C = current and F = former) is described by P followed by two subscripts, the first representing the state changed from, and the second representing the state changed to.

For the alternative scenarios, the probability of transition between the five states (N = never, C = current conventional cigarettes, T = current cMRTP, D = current dual, and F = former) is described by P followed by two subscripts, the first representing the state changed from, and the second representing the state changed to.

Table S1. Monthly tobacco transition probabilities (per million) under the null scenario.

	Initiation	Quitting	Re-Initiation
Age	P_{NC}	P_{CF}	P_{FC}
10–14	2000	500	240
15–19	3500	1500	720
20–24	2000	2000	960
25–29	1000	2000	960
30–34	500	2000	960
35–39	0	2000	960
40–44	0	2000	960
45–49	0	2000	960
50–54	0	2000	960
55–59	0	2500	1200
60–64	0	2500	1200
65–69	0	3000	1440
70–74	0	3500	1680
75–79	0	4000	1920

Table S2. Monthly tobacco transition probabilities (per million) under the "No further smoking" scenario—probabilities of initiation, cessation, and re-initiation.

Period of		Initiation			Cessation			Re-initiation		
follow up	Age	P_{NC}	P_{NT}	P_{ND}	P_{CF}	P_{TF}	P_{DF}	P_{FC}	P_{FT}	P_{FD}
1–24	10–14	0	0	0	1000000	0	0	0	0	0
	15–19	0	0	0	1000000	0	0	0	0	0
	20–24	0	0	0	1000000	0	0	0	0	0
	25–29	0	0	0	1000000	0	0	0	0	0
	30–34	0	0	0	1000000	0	0	0	0	0
	35–39	0	0	0	1000000	0	0	0	0	0
	40–44	0	0	0	1000000	0	0	0	0	0
	45–49	0	0	0	1000000	0	0	0	0	0
	50–54	0	0	0	1000000	0	0	0	0	0
	55–59	0	0	0	1000000	0	0	0	0	0
	60–64	0	0	0	1000000	0	0	0	0	0
	65–69	0	0	0	1000000	0	0	0	0	0

	70–74	0	0	0	1000000	0	0	0	0	0
	75–79	0	0	0	1000000	0	0	0	0	0
25+	10–14	0	0	0	1000000	0	0	0	0	0
	15–19	0	0	0	1000000	0	0	0	0	0
	20–24	0	0	0	1000000	0	0	0	0	0
	25–29	0	0	0	1000000	0	0	0	0	0
	30–34	0	0	0	1000000	0	0	0	0	0
	35–39	0	0	0	1000000	0	0	0	0	0
	40–44	0	0	0	1000000	0	0	0	0	0
	45–49	0	0	0	1000000	0	0	0	0	0
	50–54	0	0	0	1000000	0	0	0	0	0
	55–59	0	0	0	1000000	0	0	0	0	0
	60–64	0	0	0	1000000	0	0	0	0	0
	65–69	0	0	0	1000000	0	0	0	0	0
	70–74	0	0	0	1000000	0	0	0	0	0
	75–79	0	0	0	1000000	0	0	0	0	0

Probabilities of product switching

**Period of follow up**	**Switching between current tobacco use groups**						
	**Age**	P_{CT}	P_{CD}	P_{TC}	P_{TD}	P_{DC}	P_{DT}
Any	10–14	0	0	0	0	0	0
	15–19	0	0	0	0	0	0
	20–24	0	0	0	0	0	0
	25–29	0	0	0	0	0	0
	30–34	0	0	0	0	0	0
	35–39	0	0	0	0	0	0
	40–44	0	0	0	0	0	0
	45–49	0	0	0	0	0	0
	50–54	0	0	0	0	0	0
	55–59	0	0	0	0	0	0
	60–64	0	0	0	0	0	0
	65–69	0	0	0	0	0	0
	70–74	0	0	0	0	0	0
	75–79	0	0	0	0	0	0

Table S3. Monthly tobacco transition probabilities (per million) under the "Smoking totally replaced by cMRTP use" scenario—probabilities of initiation, cessation, and re-initiation.

**Period of**		**Initiation**			**Cessation**			**Re-initiation**		
**follow up**	**Age**	P_{NC}	P_{NT}	P_{ND}	P_{CF}	P_{TF}	P_{DF}	P_{FC}	P_{FT}	P_{FD}
1–24	10–14	0	2000	0	0	0	0	0	240	0
	15–19	0	3500	0	0	0	0	0	720	0
	20–24	0	2000	0	0	0	0	0	960	0
	25–29	0	1000	0	0	0	0	0	960	0
	30–34	0	500	0	0	0	0	0	960	0
	35–39	0	0	0	0	0	0	0	960	0
	40–44	0	0	0	0	0	0	0	960	0
	45–49	0	0	0	0	0	0	0	960	0
	50–54	0	0	0	0	0	0	0	960	0
	55–59	0	0	0	0	0	0	0	1200	0
	60–64	0	0	0	0	0	0	0	1200	0
	65–69	0	0	0	0	0	0	0	1440	0
	70–74	0	0	0	0	0	0	0	1680	0
	75–79	0	0	0	0	0	0	0	1920	0
25+	10–14	0	2000	0	0	0	0	0	240	0
	15–19	0	3500	0	0	0	0	0	720	0
	20–24	0	2000	0	0	0	0	0	960	0
	25–29	0	1000	0	0	0	0	0	960	0
	30–34	0	500	0	0	0	0	0	960	0
	35–39	0	0	0	0	0	0	0	960	0
	40–44	0	0	0	0	0	0	0	960	0
	45–49	0	0	0	0	0	0	0	960	0
	50–54	0	0	0	0	0	0	0	960	0
	55–59	0	0	0	0	0	0	0	1200	0
	60–64	0	0	0	0	0	0	0	1200	0
	65–69	0	0	0	0	0	0	0	1440	0
	70–74	0	0	0	0	0	0	0	1680	0

	75–79	0	0	0	0	0	0	0	1920	0

Probabilities of product switching

Period of follow up	Age	Switching between current tobacco use groups P_{CT}	P_{CD}	P_{TC}	P_{TD}	P_{DC}	P_{DT}
Any	10–14	1000000	0	0	0	0	1000000
	15–19	1000000	0	0	0	0	1000000
	20–24	1000000	0	0	0	0	1000000
	25–29	1000000	0	0	0	0	1000000
	30–34	1000000	0	0	0	0	1000000
	35–39	1000000	0	0	0	0	1000000
	40–44	1000000	0	0	0	0	1000000
	45–49	1000000	0	0	0	0	1000000
	50–54	1000000	0	0	0	0	1000000
	55–59	1000000	0	0	0	0	1000000
	60–64	1000000	0	0	0	0	1000000
	65–69	1000000	0	0	0	0	1000000
	70–74	1000000	0	0	0	0	1000000
	75–79	1000000	0	0	0	0	1000000

Table S4. Monthly tobacco transition probabilities (per million) under the "WHO 2025 Target" scenario—probabilities of initiation, cessation, and re-initiation.

Period of follow up	Age	Initiation P_{NC}	P_{NT}	P_{ND}	Cessation P_{CF}	P_{TF}	P_{DF}	Re–initiation P_{FC}	P_{FT}	P_{FD}
1–24	10–14	1800	0	0	1042	0	0	115	0	0
	15–19	3300	0	0	3125	0	0	346	0	0
	20–24	1800	0	0	4167	0	0	461	0	0
	25–29	800	0	0	4167	0	0	461	0	0
	30–34	300	0	0	4167	0	0	461	0	0
	35–39	0	0	0	4167	0	0	461	0	0
	40–44	0	0	0	4167	0	0	461	0	0
	45–49	0	0	0	4167	0	0	461	0	0
	50–54	0	0	0	4167	0	0	461	0	0
	55–59	0	0	0	5208	0	0	576	0	0
	60–64	0	0	0	5208	0	0	576	0	0
	65–69	0	0	0	6250	0	0	691	0	0
	70–74	0	0	0	7292	0	0	806	0	0
	75–79	0	0	0	8333	0	0	922	0	0
25+	10–14	1800	0	0	1042	0	0	115	0	0
	15–19	3300	0	0	3125	0	0	346	0	0
	20–24	1800	0	0	4167	0	0	461	0	0
	25–29	800	0	0	4167	0	0	461	0	0
	30–34	300	0	0	4167	0	0	461	0	0
	35–39	0	0	0	4167	0	0	461	0	0
	40–44	0	0	0	4167	0	0	461	0	0
	45–49	0	0	0	4167	0	0	461	0	0
	50–54	0	0	0	4167	0	0	461	0	0
	55–59	0	0	0	5208	0	0	576	0	0
	60–64	0	0	0	5208	0	0	576	0	0
	65–69	0	0	0	6250	0	0	691	0	0
	70–74	0	0	0	7292	0	0	806	0	0
	75–79	0	0	0	8333	0	0	922	0	0

Probabilities of product switching

Period of follow up	Age	Switching between current tobacco use groups P_{CT}	P_{CD}	P_{TC}	P_{TD}	P_{DC}	P_{DT}
Any	10–14	0	0	0	0	0	0
	15–19	0	0	0	0	0	0
	20–24	0	0	0	0	0	0
	25–29	0	0	0	0	0	0
	30–34	0	0	0	0	0	0
	35–39	0	0	0	0	0	0
	40–44	0	0	0	0	0	0
	45–49	0	0	0	0	0	0
	50–54	0	0	0	0	0	0
	55–59	0	0	0	0	0	0

	60–64	0	0	0	0	0	0
	65–69	0	0	0	0	0	0
	70–74	0	0	0	0	0	0
	75–79	0	0	0	0	0	0

Table S5. Monthly tobacco transition probabilities (per million) under the "WHO 2025 Projection" scenario – probabilities of initiation, cessation, and re–initiation.

**Period of follow up**	**Age**	**Initiation** P_{NC}	P_{NT}	P_{ND}	**Cessation** P_{CF}	P_{TF}	P_{DF}	**Re–initiation** P_{FC}	P_{FT}	P_{FD}
1–24	10–14	1800	0	0	833	0	0	144	0	0
	15–19	3300	0	0	2500	0	0	432	0	0
	20–24	1800	0	0	3333	0	0	576	0	0
	25–29	800	0	0	3333	0	0	576	0	0
	30–34	300	0	0	3333	0	0	576	0	0
	35–39	0	0	0	3333	0	0	576	0	0
	40–44	0	0	0	3333	0	0	576	0	0
	45–49	0	0	0	3333	0	0	576	0	0
	50–54	0	0	0	3333	0	0	576	0	0
	55–59	0	0	0	4167	0	0	720	0	0
	60–64	0	0	0	4167	0	0	720	0	0
	65–69	0	0	0	5000	0	0	864	0	0
	70–74	0	0	0	5833	0	0	1008	0	0
	75–79	0	0	0	6667	0	0	1152	0	0
25+	10–14	1800	0	0	833	0	0	144	0	0
	15–19	3300	0	0	2500	0	0	432	0	0
	20–24	1800	0	0	3333	0	0	576	0	0
	25–29	800	0	0	3333	0	0	576	0	0
	30–34	300	0	0	3333	0	0	576	0	0
	35–39	0	0	0	3333	0	0	576	0	0
	40–44	0	0	0	3333	0	0	576	0	0
	45–49	0	0	0	3333	0	0	576	0	0
	50–54	0	0	0	3333	0	0	576	0	0
	55–59	0	0	0	4167	0	0	720	0	0
	60–64	0	0	0	4167	0	0	720	0	0
	65–69	0	0	0	5000	0	0	864	0	0
	70–74	0	0	0	5833	0	0	1008	0	0
	75–79	0	0	0	6667	0	0	1152	0	0

Probabilities of product switching

**Period of follow up**	**Age**	**Switching between current tobacco use groups** P_{CT}	P_{CD}	P_{TC}	P_{TD}	P_{DC}	P_{DT}
Any	10–14	0	0	0	0	0	0
	15–19	0	0	0	0	0	0
	20–24	0	0	0	0	0	0
	25–29	0	0	0	0	0	0
	30–34	0	0	0	0	0	0
	35–39	0	0	0	0	0	0
	40–44	0	0	0	0	0	0
	45–49	0	0	0	0	0	0
	50–54	0	0	0	0	0	0
	55–59	0	0	0	0	0	0
	60–64	0	0	0	0	0	0
	65–69	0	0	0	0	0	0
	70–74	0	0	0	0	0	0
	75–79	0	0	0	0	0	0

Table S6. Monthly tobacco transition probabilities (per million) under the "cMRTP uptake case" scenario – probabilities of initiation, cessation, and re–initiation.

**Period of follow up**	**Age**	**Initiation** P_{NC}	P_{NT}	P_{ND}	**Cessation** P_{CF}	P_{TF}	P_{DF}	**Re–initiation** P_{FC}	P_{FT}	P_{FD}
1–24	10–14	1660	260	80	500	500	500	144	48	48
	15–19	2905	455	140	1500	1500	1500	432	144	144
	20–24	1660	260	80	2000	2000	2000	576	192	192
	25–29	830	130	40	2000	2000	2000	576	192	192

	30–34	415	65	20	2000	2000	2000	576	192	192
	35–39	0	0	0	2000	2000	2000	576	192	192
	40–44	0	0	0	2000	2000	2000	576	192	192
	45–49	0	0	0	2000	2000	2000	576	192	192
	50–54	0	0	0	2000	2000	2000	576	192	192
	55–59	0	0	0	2500	2500	2500	720	240	240
	60–64	0	0	0	2500	2500	2500	720	240	240
	65–69	0	0	0	3000	3000	3000	864	288	288
	70–74	0	0	0	3500	3500	3500	1008	336	336
	75–79	0	0	0	4000	4000	4000	1152	384	384
25+	10–14	1416	484	100	500	500	500	96	96	48
	15–19	2478	842	180	1500	1500	1500	288	288	144
	20–24	1416	484	100	2000	2000	2000	384	384	192
	25–29	708	230	60	2000	2000	2000	384	384	192
	30–34	354	116	30	2000	2000	2000	384	384	192
	35–39	0	0	0	2000	2000	2000	384	384	192
	40–44	0	0	0	2000	2000	2000	384	384	192
	45–49	0	0	0	2000	2000	2000	384	384	192
	50–54	0	0	0	2000	2000	2000	384	384	192
	55–59	0	0	0	2500	2500	2500	480	480	240
	60–64	0	0	0	2500	2500	2500	480	480	240
	65–69	0	0	0	3000	3000	3000	576	576	288
	70–74	0	0	0	3500	3500	3500	672	672	336
	75–79	0	0	0	4000	4000	4000	768	768	384

		Probabilities of product switching					
**Period of follow up**		Switching between current tobacco use groups					
	**Age**	P_{CT}	P_{CD}	P_{TC}	P_{TD}	P_{DC}	P_{DT}
Any	10–14	600	1	600	1	10000	400
	15–19	600	1	600	1	10000	400
	20–24	600	1	600	1	10000	400
	25–29	1200	1	600	1	10000	400
	30–34	1200	1	600	1	10000	400
	35–39	1200	1	600	1	10000	400
	40–44	1200	1	600	1	10000	400
	45–49	1200	1	600	1	10000	400
	50–54	1200	1	600	1	10000	400
	55–59	1200	1	600	1	10000	400
	60–64	1200	1	600	1	10000	400
	65–69	1200	1	600	1	10000	400
	70–74	1200	1	600	1	10000	400
	75–79	1200	1	600	1	10000	400

Table S7. Monthly tobacco transition probabilities (per million) under the "cMRTP uptake case in addition to the WHO 2025 Target" scenario – probabilities of initiation, cessation, and re–initiation.

**Period of**		**Initiation**			**Cessation**			**Re–initiation**		
**follow up**	**Age**	P_{NC}	P_{NT}	P_{ND}	P_{CF}	P_{TF}	P_{DF}	P_{FC}	P_{FT}	P_{FD}
1–24	10–14	1494	135	5	1042	1042	1042	69	23	23
	15–19	2739	406	14	3125	3125	3125	207	69	69
	20–24	1494	542	18	4167	4167	4167	276	92	92
	25–29	664	542	18	4167	4167	4167	276	92	92
	30–34	249	542	18	4167	4167	4167	276	92	92
	35–39	0	0	0	4167	4167	4167	0	0	0
	40–44	0	0	0	4167	4167	4167	0	0	0
	45–49	0	0	0	4167	4167	4167	0	0	0
	50–54	0	0	0	4167	4167	4167	0	0	0
	55–59	0	0	0	5208	5208	5208	0	0	0
	60–64	0	0	0	5208	5208	5208	0	0	0
	65–69	0	0	0	6250	6250	6250	0	0	0
	70–74	0	0	0	7292	7292	7292	0	0	0
	75–79	0	0	0	8333	8333	8333	0	0	0
25+	10–14	1274	252	6	1042	1042	1042	46	46	23
	15–19	2336	752	18	3125	3125	3125	138	138	69
	20–24	1274	1008	23	4167	4167	4167	184	184	92
	25–29	566	958	28	4167	4167	4167	184	184	92
	30–34	212	967	28	4167	4167	4167	184	184	92

	35–39	0	0	0	4167	4167	4167	0	0	0
	40–44	0	0	0	4167	4167	4167	0	0	0
	45–49	0	0	0	4167	4167	4167	0	0	0
	50–54	0	0	0	4167	4167	4167	0	0	0
	55–59	0	0	0	5208	5208	5208	0	0	0
	60–64	0	0	0	5208	5208	5208	0	0	0
	65–69	0	0	0	6250	6250	6250	0	0	0
	70–74	0	0	0	7292	7292	7292	0	0	0
	75–79	0	0	0	8333	8333	8333	0	0	0

Probabilities of product switching

Period of follow up	Age	Switching between current tobacco use groups P_{CT}	P_{CD}	P_{TC}	P_{TD}	P_{DC}	P_{DT}
Any	10–14	600	1	600	1	10000	400
	15–19	600	1	600	1	10000	400
	20–24	600	1	600	1	10000	400
	25–29	1200	1	600	1	10000	400
	30–34	1200	1	600	1	10000	400
	35–39	1200	1	600	1	10000	400
	40–44	1200	1	600	1	10000	400
	45–49	1200	1	600	1	10000	400
	50–54	1200	1	600	1	10000	400
	55–59	1200	1	600	1	10000	400
	60–64	1200	1	600	1	10000	400
	65–69	1200	1	600	1	10000	400
	70–74	1200	1	600	1	10000	400
	75–79	1200	1	600	1	10000	400

Table S8. Monthly tobacco transition probabilities (per million) under the "cMRTP uptake case in addition to the WHO 2025 Projection" scenario – probabilities of initiation, cessation, and re–initiation.

Period of		Initiation			Cessation			Re–initiation		
follow up	Age	P_{NC}	P_{NT}	P_{ND}	P_{CF}	P_{TF}	P_{DF}	P_{FC}	P_{FT}	P_{FD}
1–24	10–14	1494	234	72	833	833	833	86	29	29
	15–19	2739	429	132	2500	2500	2500	259	86	86
	20–24	1494	234	72	3333	3333	3333	346	115	115
	25–29	664	104	32	3333	3333	3333	346	115	115
	30–34	249	39	12	3333	3333	3333	346	115	115
	35–39	0	0	0	3333	3333	3333	346	115	115
	40–44	0	0	0	3333	3333	3333	346	115	115
	45–49	0	0	0	3333	3333	3333	346	115	115
	50–54	0	0	0	3333	3333	3333	346	115	115
	55–59	0	0	0	4167	4167	4167	432	144	144
	60–64	0	0	0	4167	4167	4167	432	144	144
	65–69	0	0	0	5000	5000	5000	518	173	173
	70–74	0	0	0	5833	5833	5833	605	202	202
	75–79	0	0	0	6667	6667	6667	691	230	230
25+	10–14	1274	436	90	833	833	833	58	58	29
	15–19	2336	794	170	2500	2500	2500	173	173	86
	20–24	1274	436	90	3333	3333	3333	230	230	115
	25–29	566	184	48	3333	3333	3333	230	230	115
	30–34	212	70	18	3333	3333	3333	230	230	115
	35–39	0	0	0	3333	3333	3333	230	230	115
	40–44	0	0	0	3333	3333	3333	230	230	115
	45–49	0	0	0	3333	3333	3333	230	230	115
	50–54	0	0	0	3333	3333	3333	230	230	115
	55–59	0	0	0	4167	4167	4167	288	288	144
	60–64	0	0	0	4167	4167	4167	288	288	144
	65–69	0	0	0	5000	5000	5000	346	346	173
	70–74	0	0	0	5833	5833	5833	403	403	202
	75–79	0	0	0	6667	6667	6667	461	461	230

Probabilities of product switching

Period of follow up	Age	Switching between current tobacco use groups P_{CT}	P_{CD}	P_{TC}	P_{TD}	P_{DC}	P_{DT}
Any	10–14	600	1	600	1	10000	400

	15–19	600	1	600	1	10000	400
	20–24	600	1	600	1	10000	400
	25–29	1200	1	600	1	10000	400
	30–34	1200	1	600	1	10000	400
	35–39	1200	1	600	1	10000	400
	40–44	1200	1	600	1	10000	400
	45–49	1200	1	600	1	10000	400
	50–54	1200	1	600	1	10000	400
	55–59	1200	1	600	1	10000	400
	60–64	1200	1	600	1	10000	400
	65–69	1200	1	600	1	10000	400
	70–74	1200	1	600	1	10000	400
	75–79	1200	1	600	1	10000	400

Table S9. Monthly tobacco transition probabilities (per million) under the "Extreme increase in dual use" scenario – probabilities of initiation, cessation, and re–initiation.

**Period of follow up**	**Age**	**Initiation**			**Cessation**			**Re–initiation**		
		P_{NC}	P_{NT}	P_{ND}	P_{CF}	P_{TF}	P_{DF}	P_{FC}	P_{FT}	P_{FD}
1–24	10–14	1660	260	80	500	500	500	144	48	48
	15–19	2905	455	140	1500	1500	1500	432	144	144
	20–24	1660	260	80	2000	2000	2000	576	192	192
	25–29	830	130	40	2000	2000	2000	576	192	192
	30–34	415	65	20	2000	2000	2000	576	192	192
	35–39	0	0	0	2000	2000	2000	576	192	192
	40–44	0	0	0	2000	2000	2000	576	192	192
	45–49	0	0	0	2000	2000	2000	576	192	192
	50–54	0	0	0	2000	2000	2000	576	192	192
	55–59	0	0	0	2500	2500	2500	720	240	240
	60–64	0	0	0	2500	2500	2500	720	240	240
	65–69	0	0	0	3000	3000	3000	864	288	288
	70–74	0	0	0	3500	3500	3500	1008	336	336
	75–79	0	0	0	4000	4000	4000	1152	384	384
		1416	484	100	500	500	500	96	96	48
25+	10–14	2478	842	180	1500	1500	1500	288	288	144
	15–19	1416	484	100	2000	2000	2000	384	384	192
	20–24	708	230	60	2000	2000	2000	384	384	192
	25–29	354	116	30	2000	2000	2000	384	384	192
	30–34	0	0	0	2000	2000	2000	384	384	192
	35–39	0	0	0	2000	2000	2000	384	384	192
	40–44	0	0	0	2000	2000	2000	384	384	192
	45–49	0	0	0	2000	2000	2000	384	384	192
	50–54	0	0	0	2500	2500	2500	480	480	240
	55–59	0	0	0	2500	2500	2500	480	480	240
	60–64	0	0	0	3000	3000	3000	576	576	288
	65–69	0	0	0	3500	3500	3500	672	672	336
	70–74	0	0	0	4000	4000	4000	768	768	384
	75–79	1660	260	80	500	500	500	144	48	48

Probabilities of product switching

**Period of follow up**	**Age**	**Switching between current tobacco use groups**					
		P_{CT}	P_{CD}	P_{TC}	P_{TD}	P_{DC}	P_{DT}
Any	10–14	600	313	6000	100000	10000	0
	15–19	600	313	6000	100000	10000	0
	20–24	600	313	6000	100000	10000	0
	25–29	1200	625	6000	100000	10000	0
	30–34	1200	625	6000	100000	10000	0
	35–39	1200	625	6000	100000	10000	0
	40–44	1200	625	6000	100000	10000	0
	45–49	1200	625	6000	100000	10000	0
	50–54	1200	625	6000	100000	10000	0
	55–59	1200	625	6000	100000	10000	0
	60–64	1200	625	6000	100000	10000	0
	65–69	1200	625	6000	100000	10000	0
	70–74	1200	625	6000	100000	10000	0
	75–79	1200	625	6000	100000	10000	0

healthcare

MDPI

Smoking Prevalence Over the Simulation Period in the Alternative Scenarios

Table S10. No further smoking.

Sex	Age	Scenario	Year	Never smokers (%)	Current smokers (%)	Former smokers (%)	Scenario	Never smokers (%)	Current smokers (%)	cMRTP users (%)	Dual users (%)	Former smokers (%)
**Male**	All ages	null	1990	47.7	26.6	25.6	alternative	47.7	26.6	0	0	25.6
	All ages	null	1991	47.1	27.0	25.9	alternative	47.8	0	0	0	52.2
	All ages	null	1992	46.6	27.4	26.0	alternative	47.9	0	0	0	52.1
	All ages	null	1993	45.8	28.1	26.1	alternative	48.0	0	0	0	52.0
	All ages	null	1994	45.3	28.2	26.4	alternative	48.1	0	0	0	51.9
	All ages	null	1995	44.9	28.3	26.8	alternative	48.3	0	0	0	51.7
	All ages	null	1996	44.6	28.6	26.8	alternative	48.5	0	0	0	51.5
	All ages	null	1997	44.3	28.7	27.0	alternative	48.7	0	0	0	51.3
	All ages	null	1998	44.0	28.7	27.2	alternative	49.0	0	0	0	51.0
	All ages	null	1999	43.8	28.8	27.5	alternative	49.2	0	0	0	50.8
	All ages	null	2000	43.6	28.6	27.7	alternative	49.4	0	0	0	50.6
	All ages	null	2001	43.5	28.6	27.9	alternative	49.7	0	0	0	50.3
	All ages	null	2002	43.5	28.6	28.0	alternative	50.0	0	0	0	50.0
	All ages	null	2003	43.4	28.7	27.9	alternative	50.2	0	0	0	49.8
	All ages	null	2004	43.4	28.7	27.9	alternative	50.5	0	0	0	49.5
	All ages	null	2005	43.4	28.4	28.2	alternative	50.7	0	0	0	49.3
	All ages	null	2006	43.5	28.2	28.4	alternative	51.0	0	0	0	49.0
	All ages	null	2007	43.5	28.2	28.3	alternative	51.3	0	0	0	48.7
	All ages	null	2008	43.6	27.8	28.6	alternative	51.5	0	0	0	48.5
	All ages	null	2009	43.8	27.7	28.5	alternative	51.9	0	0	0	48.1
	All ages	null	2010	44.0	27.6	28.3	alternative	52.3	0	0	0	47.7
**Female**	All ages	null	1990	59.2	21.6	19.3	alternative	59.2	21.6	0	0	19.3

	All ages	null	1991	58.3	22.2	19.5	alternative	59.1	0	0	0	40.9
	All ages	null	1992	57.4	22.7	19.9	alternative	59.0	0	0	0	41.0
	All ages	null	1993	56.6	23.2	20.2	alternative	58.9	0	0	0	41.1
	All ages	null	1994	56.1	23.4	20.5	alternative	58.9	0	0	0	41.1
	All ages	null	1995	55.3	23.9	20.7	alternative	58.8	0	0	0	41.2
	All ages	null	1996	54.8	24.0	21.1	alternative	58.8	0	0	0	41.2
	All ages	null	1997	54.3	24.4	21.3	alternative	58.9	0	0	0	41.1
	All ages	null	1998	53.9	24.5	21.7	alternative	58.9	0	0	0	41.1
	All ages	null	1999	53.6	24.6	21.8	alternative	59.0	0	0	0	41.0
	All ages	null	2000	53.4	24.4	22.2	alternative	59.0	0	0	0	41.0
	All ages	null	2001	53.1	24.4	22.5	alternative	59.1	0	0	0	40.9
	All ages	null	2002	52.8	24.6	22.5	alternative	59.1	0	0	0	40.9
	All ages	null	2003	52.6	24.7	22.6	alternative	59.2	0	0	0	40.8
	All ages	null	2004	52.5	24.4	23.0	alternative	59.3	0	0	0	40.7
	All ages	null	2005	52.4	24.5	23.1	alternative	59.4	0	0	0	40.6
	All ages	null	2006	52.3	24.6	23.1	alternative	59.6	0	0	0	40.4
	All ages	null	2007	52.3	24.2	23.4	alternative	59.8	0	0	0	40.2
	All ages	null	2008	52.3	24.1	23.6	alternative	59.9	0	0	0	40.1
	All ages	null	2009	52.2	24.1	23.7	alternative	60.0	0	0	0	40.0
	All ages	null	2010	52.3	23.7	24.0	alternative	60.2	0	0	0	39.8

Table S11. Smoking totally replaced by cMRTP use.

Sex	Age	Scenario	Year #	Never smoker (%)	Current smoker (%)	Former smoker (%)	Scenario	Never smoker (%)	Current smoker (%)	cMRTP users (%)	Dual users (%)	Former smoker (%)
**Male**	All ages	null	1990	47.7	26.6	25.6	alternative	47.7	26.6	0	0	25.6
	All ages	null	1991	47.1	27.0	25.9	alternative	46.9	0	28.0	0	25.2
	All ages	null	1992	46.6	27.4	26.0	alternative	46.5	0	28.0	0	25.5
	All ages	null	1993	45.8	28.1	26.1	alternative	46.0	0	28.0	0	25.9

	All ages	null	1994	45.3	28.2	26.4	alternative	45.6	0	28.0	0	26.0
	All ages	null	1995	44.9	28.3	26.8	alternative	45.0	0	29.0	0	26.3
	All ages	null	1996	44.6	28.6	26.8	alternative	44.5	0	29.0	0	26.4
	All ages	null	1997	44.3	28.7	27.0	alternative	44.1	0	29.0	0	26.5
	All ages	null	1998	44.0	28.7	27.2	alternative	43.9	0	29.0	0	26.6
	All ages	null	1999	43.8	28.8	27.5	alternative	43.6	0	29.0	0	27.0
	All ages	null	2000	43.6	28.6	27.7	alternative	43.4	0	29.0	0	27.1
	All ages	null	2001	43.5	28.6	27.9	alternative	43.3	0	29.0	0	27.3
	All ages	null	2002	43.5	28.6	28.0	alternative	43.3	0	29.0	0	27.4
	All ages	null	2003	43.4	28.7	27.9	alternative	43.4	0	29.0	0	27.5
	All ages	null	2004	43.4	28.7	27.9	alternative	43.3	0	29.0	0	27.6
	All ages	null	2005	43.4	28.4	28.2	alternative	43.3	0	29.0	0	27.7
	All ages	null	2006	43.5	28.2	28.4	alternative	43.5	0	29.0	0	27.7
	All ages	null	2007	43.5	28.2	28.3	alternative	43.6	0	28.0	0	28.1
	All ages	null	2008	43.6	27.8	28.6	alternative	43.6	0	28.0	0	28.3
	All ages	null	2009	43.8	27.7	28.5	alternative	43.9	0	28.0	0	28.4
	All ages	null	2010	44.0	27.6	28.3	alternative	44.1	0	27.4	0	28.4
**Female**	All ages	null	1990	59.2	21.6	19.3	alternative	59.2	21.6	0	0	19.3
	All ages	null	1991	58.3	22.2	19.5	alternative	58.5	0	22.7	0	18.9
	All ages	null	1992	57.4	22.7	19.9	alternative	57.7	0	23.0	0	19.3
	All ages	null	1993	56.6	23.2	20.2	alternative	56.9	0	23.6	0	19.5
	All ages	null	1994	56.1	23.4	20.5	alternative	56.4	0	23.8	0	19.8
	All ages	null	1995	55.3	23.9	20.7	alternative	55.6	0	24.4	0	20.0
	All ages	null	1996	54.8	24.0	21.1	alternative	55.1	0	24.7	0	20.2
	All ages	null	1997	54.3	24.4	21.3	alternative	54.8	0	24.7	0	20.5
	All ages	null	1998	53.9	24.5	21.7	alternative	54.4	0	24.8	0	20.8
	All ages	null	1999	53.6	24.6	21.8	alternative	54.1	0	24.9	0	21.0
	All ages	null	2000	53.4	24.4	22.2	alternative	53.8	0	25.0	0	21.2
	All ages	null	2001	53.1	24.4	22.5	alternative	53.4	0	25.1	0	21.5

	All ages	null	2002	52.8	24.6	22.5	alternative	53.3	0	25.0	0	21.7
	All ages	null	2003	52.6	24.7	22.6	alternative	53.1	0	24.9	0	22.0
	All ages	null	2004	52.5	24.4	23.0	alternative	53.0	0	24.8	0	22.3
	All ages	null	2005	52.4	24.5	23.1	alternative	52.8	0	24.6	0	22.6
	All ages	null	2006	52.3	24.6	23.1	alternative	52.7	0	24.4	0	22.8
	All ages	null	2007	52.3	24.2	23.4	alternative	52.7	0	24.2	0	23.1
	All ages	null	2008	52.3	24.1	23.6	alternative	52.6	0	23.9	0	23.4
	All ages	null	2009	52.2	24.1	23.7	alternative	52.5	0	23.9	0	23.6
	All ages	null	2010	52.3	23.7	24.0	alternative	52.6	0	23.6	0	23.8

Table S12. WHO 2025 Target.

**Sex**	**Age**	**Scenario**	**Year #**	**Never smoker (%)**	**Current smoker (%)**	**Former smoker (%)**	**Scenario**	**Never smoker (%)**	**Current smoker (%)**	**cMRTP users (%)**	**Dual users (%)**	**Former smoker (%)**
**Male**	All ages	null	1990	47.9	26.7	25.4	alternative	47.9	26.7	0	0	25.4
	All ages	null	1991	47.2	27.1	25.6	alternative	47.4	26.3	0	0	26.4
	All ages	null	1992	46.7	27.5	25.8	alternative	47.0	25.7	0	0	27.4
	All ages	null	1993	46.3	27.8	25.9	alternative	46.7	25.0	0	0	28.3
	All ages	null	1994	45.6	28.3	26.0	alternative	46.2	24.5	0	0	29.3
	All ages	null	1995	45.0	28.9	26.0	alternative	45.5	24.3	0	0	30.1
	All ages	null	1996	44.6	29.3	26.1	alternative	45.1	23.9	0	0	30.9
	All ages	null	1997	44.3	29.1	26.5	alternative	44.8	23.5	0	0	31.7
	All ages	null	1998	44.1	29.3	26.6	alternative	44.6	23.0	0	0	32.4
	All ages	null	1999	44.0	29.0	27.0	alternative	44.4	22.5	0	0	33.1
	All ages	null	2000	43.9	28.8	27.3	alternative	44.4	21.9	0	0	33.8
	All ages	null	2001	43.8	28.8	27.4	alternative	44.3	21.3	0	0	34.4
	All ages	null	2002	43.8	28.7	27.6	alternative	44.4	20.6	0	0	35.0
	All ages	null	2003	43.9	28.3	27.8	alternative	44.5	19.9	0	0	35.6
	All ages	null	2004	43.9	28.3	27.8	alternative	44.6	19.3	0	0	36.1

	All ages	null	2005	44.1	28.1	27.9	alternative	44.8	18.5	0	0	36.7
	All ages	null	2006	44.2	27.9	27.9	alternative	45.0	17.6	0	0	37.5
	All ages	null	2007	44.3	27.9	27.8	alternative	45.1	17.1	0	0	37.8
	All ages	null	2008	44.4	27.5	28.1	alternative	45.3	16.6	0	0	38.1
	All ages	null	2009	44.6	27.1	28.3	alternative	45.5	16.1	0	0	38.4
	All ages	null	2010	44.8	26.8	28.3	alternative	45.8	15.7	0	0	38.5
**Female**	All ages	null	1990	59.8	22.0	18.3	alternative	59.8	22.0	0	0	18.3
	All ages	null	1991	59.0	22.3	18.7	alternative	59.1	21.6	0	0	19.3
	All ages	null	1992	58.4	22.7	18.9	alternative	58.5	21.2	0	0	20.4
	All ages	null	1993	57.9	22.8	19.3	alternative	57.9	20.6	0	0	21.5
	All ages	null	1994	57.3	23.1	19.6	alternative	57.3	20.1	0	0	22.6
	All ages	null	1995	56.7	23.4	19.9	alternative	56.7	19.9	0	0	23.4
	All ages	null	1996	56.3	23.7	19.9	alternative	56.3	19.5	0	0	24.3
	All ages	null	1997	55.9	23.8	20.3	alternative	55.9	19.0	0	0	25.1
	All ages	null	1998	55.5	23.9	20.6	alternative	55.4	18.7	0	0	25.9
	All ages	null	1999	55.1	24.3	20.7	alternative	55.0	18.4	0	0	26.6
	All ages	null	2000	54.8	24.4	20.7	alternative	54.8	17.8	0	0	27.3
	All ages	null	2001	54.6	24.4	21.0	alternative	54.7	17.4	0	0	27.9
	All ages	null	2002	54.3	24.5	21.2	alternative	54.4	17.0	0	0	28.6
	All ages	null	2003	54.2	24.5	21.3	alternative	54.4	16.8	0	0	28.8
	All ages	null	2004	54.0	24.3	21.7	alternative	54.2	16.4	0	0	29.3
	All ages	null	2005	53.9	24.3	21.8	alternative	54.1	16.0	0	0	29.9
	All ages	null	2006	53.8	24.2	22.0	alternative	54.1	15.6	0	0	30.2
	All ages	null	2007	53.8	23.9	22.3	alternative	54.2	14.9	0	0	31.0
	All ages	null	2008	53.6	23.6	22.7	alternative	54.2	14.3	0	0	31.5
	All ages	null	2009	53.7	23.5	22.8	alternative	54.3	13.8	0	0	31.9
	All ages	null	2010	53.7	23.5	22.8	alternative	54.4	13.3	0	0	32.3

Table S13. WHO 2025 Projection.

Sex	Age	Scenario	Year #	Never smoker (%)	Current smoker (%)	Former smoker (%)	Scenario	Never smoker (%)	Current smoker (%)	cMRTP users (%)	Dual users (%)	Former smoker (%)
**Male**	All ages	null	1990	47.9	26.7	25.4	alternative	47.9	26.7	0	0	25.4
	All ages	null	1991	47.2	27.1	25.6	alternative	47.4	26.5	0	0	26.1
	All ages	null	1992	46.7	27.5	25.8	alternative	47.0	26.3	0	0	26.8
	All ages	null	1993	46.3	27.8	25.9	alternative	46.7	26.0	0	0	27.3
	All ages	null	1994	45.6	28.3	26.0	alternative	46.2	25.8	0	0	28.1
	All ages	null	1995	45.0	28.9	26.0	alternative	45.5	25.8	0	0	28.7
	All ages	null	1996	44.6	29.3	26.1	alternative	45.1	25.7	0	0	29.2
	All ages	null	1997	44.3	29.1	26.5	alternative	44.8	25.4	0	0	29.8
	All ages	null	1998	44.1	29.3	26.6	alternative	44.6	25.1	0	0	30.2
	All ages	null	1999	44.0	29.0	27.0	alternative	44.4	24.7	0	0	30.9
	All ages	null	2000	43.9	28.8	27.3	alternative	44.4	24.2	0	0	31.5
	All ages	null	2001	43.8	28.8	27.4	alternative	44.3	23.7	0	0	32.0
	All ages	null	2002	43.8	28.7	27.6	alternative	44.4	23.2	0	0	32.5
	All ages	null	2003	43.9	28.3	27.8	alternative	44.5	22.6	0	0	32.9
	All ages	null	2004	43.9	28.3	27.8	alternative	44.6	22.0	0	0	33.5
	All ages	null	2005	44.1	28.1	27.9	alternative	44.8	21.3	0	0	33.9
	All ages	null	2006	44.2	27.9	27.9	alternative	45.0	20.5	0	0	34.5
	All ages	null	2007	44.3	27.9	27.8	alternative	45.1	20.1	0	0	34.8
	All ages	null	2008	44.4	27.5	28.1	alternative	45.3	19.6	0	0	35.1
	All ages	null	2009	44.6	27.1	28.3	alternative	45.5	19.2	0	0	35.3
	All ages	null	2010	44.8	26.8	28.3	alternative	45.8	18.8	0	0	35.4
**Female**	All ages	null	1990	59.8	22.0	18.3	alternative	59.8	22.0	0	0	18.3
	All ages	null	1991	59.0	22.3	18.7	alternative	59.1	21.9	0	0	19.0
	All ages	null	1992	58.4	22.7	18.9	alternative	58.5	21.7	0	0	19.8
	All ages	null	1993	57.9	22.8	19.3	alternative	57.9	21.5	0	0	20.6
	All ages	null	1994	57.3	23.1	19.6	alternative	57.3	21.1	0	0	21.6
	All ages	null	1995	56.7	23.4	19.9	alternative	56.7	21.2	0	0	22.2
	All ages	null	1996	56.3	23.7	19.9	alternative	56.3	20.9	0	0	22.8

	All ages	null	1997	55.9	23.8	20.3	alternative	55.9	20.6	0	0	23.5
	All ages	null	1998	55.5	23.9	20.6	alternative	55.4	20.3	0	0	24.3
	All ages	null	1999	55.1	24.3	20.7	alternative	55.0	20.1	0	0	24.9
	All ages	null	2000	54.8	24.4	20.7	alternative	54.8	19.7	0	0	25.4
	All ages	null	2001	54.6	24.4	21.0	alternative	54.7	19.4	0	0	25.9
	All ages	null	2002	54.3	24.5	21.2	alternative	54.4	19.2	0	0	26.4
	All ages	null	2003	54.2	24.5	21.3	alternative	54.4	19.1	0	0	26.5
	All ages	null	2004	54.0	24.3	21.7	alternative	54.2	19.0	0	0	26.8
	All ages	null	2005	53.9	24.3	21.8	alternative	54.1	18.5	0	0	27.3
	All ages	null	2006	53.8	24.2	22.0	alternative	54.1	18.1	0	0	27.7
	All ages	null	2007	53.8	23.9	22.3	alternative	54.2	17.5	0	0	28.4
	All ages	null	2008	53.6	23.6	22.7	alternative	54.2	16.9	0	0	28.9
	All ages	null	2009	53.7	23.5	22.8	alternative	54.3	16.4	0	0	29.3
	All ages	null	2010	53.7	23.5	22.8	alternative	54.4	16.1	0	0	29.6

Table S14. cMRTP uptake case.

Sex	Age	Scenario	Year #	Never smoker (%)	Current smoker (%)	Former smoker (%)	Scenario	Never smoker (%)	Current smoker (%)	cMRTP users (%)	Dual users (%)	Former smoker (%)
**Male**	All ages	null	1990	47.7	26.6	25.6	alternative	47.7	26.6	0.0	0.0	25.6
	All ages	null	1991	47.1	27.1	25.8	alternative	46.9	26.7	0.5	0.1	25.8
	All ages	null	1992	46.6	27.6	25.9	alternative	46.5	26.4	1.0	0.2	26.0
	All ages	null	1993	45.8	28.2	26.0	alternative	46.0	25.9	1.5	0.3	26.3
	All ages	null	1994	45.3	28.3	26.4	alternative	45.6	25.4	2.1	0.4	26.5
	All ages	null	1995	45.0	28.3	26.7	alternative	45.0	25.1	2.6	0.5	26.7
	All ages	null	1996	44.6	28.6	26.8	alternative	44.5	25.0	3.3	0.5	26.7
	All ages	null	1997	44.3	28.6	27.1	alternative	44.1	24.8	3.9	0.6	26.6
	All ages	null	1998	44.0	28.6	27.3	alternative	43.9	24.4	4.4	0.6	26.8
	All ages	null	1999	43.8	28.6	27.6	alternative	43.6	23.9	4.6	0.6	27.4
	All ages	null	2000	43.6	28.5	27.9	alternative	43.5	23.5	5.0	0.5	27.6

	All ages	null	2001	43.5	28.4	28.1	alternative	43.3	23.0	5.4	0.5	27.8
	All ages	null	2002	43.5	28.4	28.2	alternative	43.3	22.6	5.8	0.5	27.8
	All ages	null	2003	43.4	28.5	28.1	alternative	43.4	22.0	6.1	0.5	28.1
	All ages	null	2004	43.4	28.5	28.1	alternative	43.3	21.5	6.4	0.5	28.3
	All ages	null	2005	43.4	28.2	28.4	alternative	43.3	21.3	6.7	0.5	28.2
	All ages	null	2006	43.5	27.9	28.6	alternative	43.5	20.8	7.0	0.6	28.2
	All ages	null	2007	43.5	27.8	28.6	alternative	43.6	20.2	7.3	0.5	28.4
	All ages	null	2008	43.6	27.4	29.0	alternative	43.6	19.6	7.5	0.6	28.7
	All ages	null	2009	43.8	27.4	28.9	alternative	43.9	18.9	7.6	0.6	29.1
	All ages	null	2010	44.0	27.2	28.8	alternative	44.1	18.4	7.9	0.6	29.1
**Female**	All ages	null	1990	59.2	21.6	19.3	alternative	59.2	21.6	0.0	0.0	19.3
	All ages	null	1991	58.3	22.2	19.5	alternative	58.5	21.6	0.4	0.1	19.5
	All ages	null	1992	57.4	22.7	19.9	alternative	57.7	21.6	0.8	0.1	19.8
	All ages	null	1993	56.6	23.3	20.1	alternative	56.9	21.6	1.2	0.2	20.1
	All ages	null	1994	56.1	23.4	20.5	alternative	56.4	21.4	1.7	0.2	20.4
	All ages	null	1995	55.3	23.9	20.8	alternative	55.6	21.2	2.2	0.4	20.7
	All ages	null	1996	54.8	24.0	21.2	alternative	55.1	21.0	2.6	0.4	20.9
	All ages	null	1997	54.3	24.4	21.3	alternative	54.8	20.6	2.9	0.4	21.3
	All ages	null	1998	53.9	24.4	21.8	alternative	54.4	20.3	3.2	0.4	21.8
	All ages	null	1999	53.6	24.5	21.9	alternative	54.1	19.9	3.6	0.4	22.0
	All ages	null	2000	53.4	24.3	22.3	alternative	53.8	19.8	3.9	0.4	22.2
	All ages	null	2001	53.1	24.3	22.7	alternative	53.4	19.5	4.3	0.4	22.3
	All ages	null	2002	52.8	24.5	22.7	alternative	53.3	19.1	4.6	0.4	22.7
	All ages	null	2003	52.7	24.5	22.8	alternative	53.1	18.6	4.8	0.5	23.0
	All ages	null	2004	52.5	24.2	23.2	alternative	53.0	18.0	5.2	0.5	23.4
	All ages	null	2005	52.4	24.2	23.4	alternative	52.8	17.5	5.5	0.4	23.8
	All ages	null	2006	52.3	24.3	23.4	alternative	52.7	16.8	5.7	0.4	24.3
	All ages	null	2007	52.3	23.8	23.9	alternative	52.7	16.4	5.9	0.4	24.6
	All ages	null	2008	52.3	23.7	24.1	alternative	52.6	15.9	6.0	0.5	24.9

	All ages	null	2009	52.2	23.7	24.1	alternative	52.5	15.8	6.1	0.4	25.2
	All ages	null	2010	52.3	23.3	24.5	alternative	52.6	15.4	6.2	0.5	25.4

Table S15. cMRTP uptake case in addition to the WHO 2025 Target.

Sex	Age	Scenario	Year #	Never smoker (%)	Current smoker (%)	Former smoker (%)	Scenario	Never smoker (%)	Current smoker (%)	cMRTP users (%)	Dual users (%)	Former smoker (%)
**Male**	All ages	null	1990	47.9	26.7	25.4	alternative	47.9	26.7	0.0	0.0	25.4
	All ages	null	1991	47.2	27.1	25.6	alternative	47.3	25.7	0.5	0.0	26.5
	All ages	null	1992	46.7	27.5	25.8	alternative	46.8	24.6	0.8	0.0	27.8
	All ages	null	1993	46.3	27.8	25.9	alternative	46.4	23.5	1.3	0.0	28.7
	All ages	null	1994	45.6	28.3	26.0	alternative	45.9	22.5	1.9	0.0	29.7
	All ages	null	1995	45.0	28.9	26.0	alternative	45.1	21.7	2.4	0.0	30.7
	All ages	null	1996	44.6	29.3	26.1	alternative	44.6	20.8	2.8	0.0	31.7
	All ages	null	1997	44.3	29.1	26.5	alternative	44.1	19.8	3.3	0.0	32.7
	All ages	null	1998	44.1	29.3	26.6	alternative	43.8	18.9	3.7	0.1	33.6
	All ages	null	1999	44.0	29.0	27.0	alternative	43.3	18.1	4.0	0.0	34.5
	All ages	null	2000	43.9	28.8	27.3	alternative	43.1	17.1	4.3	0.0	35.4
	All ages	null	2001	43.8	28.8	27.4	alternative	42.9	16.2	4.6	0.1	36.2
	All ages	null	2002	43.8	28.7	27.6	alternative	42.8	15.3	4.8	0.1	36.9
	All ages	null	2003	43.9	28.3	27.8	alternative	42.8	14.7	4.9	0.1	37.6
	All ages	null	2004	43.9	28.3	27.8	alternative	42.8	14.0	5.0	0.1	38.2
	All ages	null	2005	44.1	28.1	27.9	alternative	42.8	13.2	5.0	0.1	38.9
	All ages	null	2006	44.2	27.9	27.9	alternative	42.8	12.4	5.2	0.1	39.6
	All ages	null	2007	44.3	27.9	27.8	alternative	42.9	11.8	5.3	0.1	40.0
	All ages	null	2008	44.4	27.5	28.1	alternative	42.9	11.3	5.2	0.0	40.5
	All ages	null	2009	44.6	27.1	28.3	alternative	43.0	10.8	5.2	0.1	41.0
	All ages	null	2010	44.8	26.8	28.3	alternative	43.2	10.3	5.0	0.0	41.5
**Female**	All ages	null	1990	59.8	22.0	18.3	alternative	59.8	22.0	0.0	0.0	18.3
	All ages	null	1991	59.0	22.3	18.7	alternative	59.0	21.0	0.4	0.0	19.5
	All ages	null	1992	58.4	22.7	18.9	alternative	58.3	20.2	0.9	0.0	20.6

	All ages	null	1993	57.9	22.8	19.3	alternative	57.7	19.3	1.3	0.0	21.6
	All ages	null	1994	57.3	23.1	19.6	alternative	56.9	18.6	1.8	0.0	22.6
	All ages	null	1995	56.7	23.4	19.9	alternative	56.2	18.0	2.2	0.0	23.6
	All ages	null	1996	56.3	23.7	19.9	alternative	55.6	17.2	2.7	0.0	24.5
	All ages	null	1997	55.9	23.8	20.3	alternative	55.1	16.4	3.0	0.0	25.5
	All ages	null	1998	55.5	23.9	20.6	alternative	54.5	15.8	3.3	0.0	26.4
	All ages	null	1999	55.1	24.3	20.7	alternative	54.0	15.2	3.6	0.0	27.2
	All ages	null	2000	54.8	24.4	20.7	alternative	53.7	14.4	3.8	0.0	28.1
	All ages	null	2001	54.6	24.4	21.0	alternative	53.5	13.7	3.8	0.0	28.9
	All ages	null	2002	54.3	24.5	21.2	alternative	53.0	13.0	4.1	0.0	29.8
	All ages	null	2003	54.2	24.5	21.3	alternative	52.8	12.5	4.2	0.0	30.5
	All ages	null	2004	54.0	24.3	21.7	alternative	52.6	11.9	4.3	0.0	31.2
	All ages	null	2005	53.9	24.3	21.8	alternative	52.3	11.3	4.3	0.1	32.0
	All ages	null	2006	53.8	24.2	22.0	alternative	52.2	10.7	4.3	0.0	32.8
	All ages	null	2007	53.8	23.9	22.3	alternative	52.1	10.2	4.4	0.0	33.3
	All ages	null	2008	53.6	23.6	22.7	alternative	52.0	9.7	4.5	0.0	33.8
	All ages	null	2009	53.7	23.5	22.8	alternative	52.0	9.0	4.6	0.0	34.4
	All ages	null	2010	53.7	23.5	22.8	alternative	52.1	8.6	4.5	0.0	34.9

Table S16. cMRTP uptake case in addition to the WHO 2025 Projection.

**Sex**	**Age**	**Scenario**	**Year #**	**Never smoker (%)**	**Current smoker (%)**	**Former smoker (%)**	**Scenario**	**Never smoker (%)**	**Current smoker (%)**	**cMRTP users (%)**	**Dual users (%)**	**Former smoker (%)**
**Male**	All ages	null	1990	47.9	26.7	25.4	alternative	47.9	26.7	0.0	0.0	25.4
	All ages	null	1991	47.2	27.1	25.6	alternative	47.4	26.1	0.4	0.1	26.0
	All ages	null	1992	46.7	27.5	25.8	alternative	47.0	25.4	0.7	0.2	26.9
	All ages	null	1993	46.3	27.8	25.9	alternative	46.7	24.5	1.2	0.3	27.4
	All ages	null	1994	45.6	28.3	26.0	alternative	46.2	23.8	1.7	0.2	28.1
	All ages	null	1995	45.0	28.9	26.0	alternative	45.5	23.3	2.2	0.3	28.7
	All ages	null	1996	44.6	29.3	26.1	alternative	45.1	22.6	2.6	0.3	29.4
	All ages	null	1997	44.3	29.1	26.5	alternative	44.8	21.9	3.1	0.3	29.8

	All ages	null	1998	44.1	29.3	26.6	alternative	44.6	21.2	3.5	0.4	30.3
	All ages	null	1999	44.0	29.0	27.0	alternative	44.4	20.5	3.8	0.4	30.9
	All ages	null	2000	43.9	28.8	27.3	alternative	44.4	19.7	4.1	0.3	31.5
	All ages	null	2001	43.8	28.8	27.4	alternative	44.3	19.1	4.4	0.3	31.9
	All ages	null	2002	43.8	28.7	27.6	alternative	44.4	18.4	4.6	0.3	32.3
	All ages	null	2003	43.9	28.3	27.8	alternative	44.5	17.7	4.7	0.4	32.8
	All ages	null	2004	43.9	28.3	27.8	alternative	44.6	17.0	4.8	0.4	33.2
	All ages	null	2005	44.1	28.1	27.9	alternative	44.8	16.2	4.9	0.4	33.8
	All ages	null	2006	44.2	27.9	27.9	alternative	45.0	15.3	5.0	0.4	34.3
	All ages	null	2007	44.3	27.9	27.8	alternative	45.1	14.8	5.2	0.4	34.4
	All ages	null	2008	44.4	27.5	28.1	alternative	45.3	14.3	5.3	0.4	34.6
	All ages	null	2009	44.6	27.1	28.3	alternative	45.5	13.9	5.3	0.4	34.9
	All ages	null	2010	44.8	26.8	28.3	alternative	45.8	13.5	5.4	0.4	35.0
**Female**	All ages	null	1990	59.8	22.0	18.3	alternative	59.8	22.0	0.0	0.0	18.3
	All ages	null	1991	59.0	22.3	18.7	alternative	59.1	21.5	0.3	0.1	19.0
	All ages	null	1992	58.4	22.7	18.9	alternative	58.5	20.9	0.8	0.1	19.8
	All ages	null	1993	57.9	22.8	19.3	alternative	57.9	20.2	1.2	0.2	20.6
	All ages	null	1994	57.3	23.1	19.6	alternative	57.3	19.5	1.6	0.2	21.4
	All ages	null	1995	56.7	23.4	19.9	alternative	56.7	19.1	1.9	0.2	22.1
	All ages	null	1996	56.3	23.7	19.9	alternative	56.3	18.5	2.3	0.2	22.7
	All ages	null	1997	55.9	23.8	20.3	alternative	55.9	17.9	2.6	0.2	23.4
	All ages	null	1998	55.5	23.9	20.6	alternative	55.4	17.5	2.8	0.2	24.1
	All ages	null	1999	55.1	24.3	20.7	alternative	55.0	17.0	3.1	0.2	24.6
	All ages	null	2000	54.8	24.4	20.7	alternative	54.8	16.4	3.4	0.2	25.1
	All ages	null	2001	54.6	24.4	21.0	alternative	54.7	15.9	3.5	0.3	25.6
	All ages	null	2002	54.3	24.5	21.2	alternative	54.4	15.3	3.7	0.3	26.3
	All ages	null	2003	54.2	24.5	21.3	alternative	54.4	14.9	3.8	0.3	26.6
	All ages	null	2004	54.0	24.3	21.7	alternative	54.2	14.5	4.0	0.3	27.0
	All ages	null	2005	53.9	24.3	21.8	alternative	54.1	14.0	4.1	0.3	27.4

	All ages	null	2006	53.8	24.2	22.0	alternative	54.1	13.6	4.2	0.3	27.8
	All ages	null	2007	53.8	23.9	22.3	alternative	54.2	12.9	4.3	0.3	28.4
	All ages	null	2008	53.6	23.6	22.7	alternative	54.2	12.4	4.3	0.3	28.8
	All ages	null	2009	53.7	23.5	22.8	alternative	54.3	11.9	4.4	0.3	29.2
	All ages	null	2010	53.7	23.5	22.8	alternative	54.4	11.3	4.4	0.3	29.5

Table S17. Extreme increase in dual use.

Sex	Age	Scenario	Year #	Never smoker (%)	Current smoker (%)	Former smoker (%)	Scenario	Never smoker (%)	Current smoker (%)	cMRTP users (%)	Dual users (%)	Former smoker (%)
**Male**	All ages	null	1990	47.7	26.6	25.6	alternative	47.7	26.6	0.0	0.0	25.6
	All ages	null	1991	47.1	27.1	25.8	alternative	46.9	26.5	0.5	0.2	25.9
	All ages	null	1992	46.6	27.6	25.9	alternative	46.5	26.0	0.6	0.9	26.1
	All ages	null	1993	45.8	28.2	26.0	alternative	46.0	25.4	0.8	1.4	26.5
	All ages	null	1994	45.3	28.3	26.4	alternative	45.6	24.9	0.8	2.0	26.7
	All ages	null	1995	44.9	28.3	26.7	alternative	45.0	24.6	1.0	2.5	26.8
	All ages	null	1996	44.6	28.6	26.8	alternative	44.5	24.4	1.4	2.9	26.7
	All ages	null	1997	44.3	28.6	27.1	alternative	44.1	24.5	1.4	3.3	26.6
	All ages	null	1998	44.0	28.6	27.3	alternative	43.9	24.2	1.5	3.6	26.8
	All ages	null	1999	43.8	28.6	27.6	alternative	43.6	24.1	1.4	3.6	27.3
	All ages	null	2000	43.6	28.5	27.9	alternative	43.4	23.8	1.7	3.6	27.5
	All ages	null	2001	43.5	28.4	28.1	alternative	43.3	23.6	1.7	3.8	27.6
	All ages	null	2002	43.5	28.4	28.2	alternative	43.3	23.3	1.9	3.8	27.7
	All ages	null	2003	43.4	28.5	28.0	alternative	43.4	22.8	1.9	3.9	28.0
	All ages	null	2004	43.4	28.5	28.1	alternative	43.3	22.6	2.1	4.1	28.0
	All ages	null	2005	43.4	28.2	28.4	alternative	43.3	22.5	2.0	4.1	28.0
	All ages	null	2006	43.5	27.9	28.6	alternative	43.5	22.3	2.1	4.1	28.1
	All ages	null	2007	43.5	27.8	28.6	alternative	43.6	22.0	2.4	3.9	28.1
	All ages	null	2008	43.6	27.4	29.0	alternative	43.6	21.5	2.5	4.0	28.4

	All ages	null	2009	43.8	27.4	28.9	alternative	43.9	21.0	2.3	4.2	28.6
	All ages	null	2010	44.0	27.2	28.8	alternative	44.1	20.6	2.6	4.1	28.4
**Female**	All ages	null	1990	59.2	21.6	19.3	alternative	59.2	21.6	0.0	0.0	19.3
	All ages	null	1991	58.3	22.2	19.5	alternative	58.5	21.4	0.4	0.3	19.4
	All ages	null	1992	57.4	22.7	19.9	alternative	57.7	21.3	0.5	0.8	19.7
	All ages	null	1993	56.6	23.3	20.1	alternative	56.9	21.3	0.7	1.1	20.0
	All ages	null	1994	56.1	23.4	20.5	alternative	56.4	21.1	0.8	1.4	20.2
	All ages	null	1995	55.3	23.9	20.8	alternative	55.6	21.0	1.0	1.9	20.5
	All ages	null	1996	54.8	24.0	21.2	alternative	55.1	20.8	1.0	2.2	20.9
	All ages	null	1997	54.3	24.4	21.3	alternative	54.8	20.4	1.0	2.6	21.2
	All ages	null	1998	53.9	24.4	21.8	alternative	54.4	20.2	1.1	2.8	21.5
	All ages	null	1999	53.6	24.5	21.9	alternative	54.1	19.9	1.2	3.1	21.7
	All ages	null	2000	53.4	24.3	22.3	alternative	53.8	19.8	1.4	3.2	21.9
	All ages	null	2001	53.1	24.3	22.7	alternative	53.4	19.7	1.5	3.4	22.0
	All ages	null	2002	52.8	24.4	22.7	alternative	53.3	19.5	1.5	3.6	22.2
	All ages	null	2003	52.6	24.5	22.8	alternative	53.1	19.1	1.5	3.6	22.6
	All ages	null	2004	52.5	24.2	23.2	alternative	53.0	18.8	1.6	3.7	23.0
	All ages	null	2005	52.4	24.2	23.4	alternative	52.8	18.5	1.9	3.6	23.2
	All ages	null	2006	52.3	24.3	23.4	alternative	52.7	18.1	1.8	3.7	23.7
	All ages	null	2007	52.3	23.8	23.9	alternative	52.7	18.0	1.8	3.6	23.9
	All ages	null	2008	52.3	23.7	24.1	alternative	52.6	17.8	1.8	3.7	24.1
	All ages	null	2009	52.2	23.7	24.1	alternative	52.5	17.7	1.9	3.4	24.4
	All ages	null	2010	52.3	23.3	24.5	alternative	52.6	17.4	2.1	3.4	24.5
